# Supplementary figures and images for: A hydrogel model of the human blood-brain barrier using differentiated stem cells
Source: PLoS One. 2023 Apr 4;18(4):e0283954. doi: 10.1371/journal.pone.0283954 (PMC10072488; doi:10.1371/journal.pone.0283954)

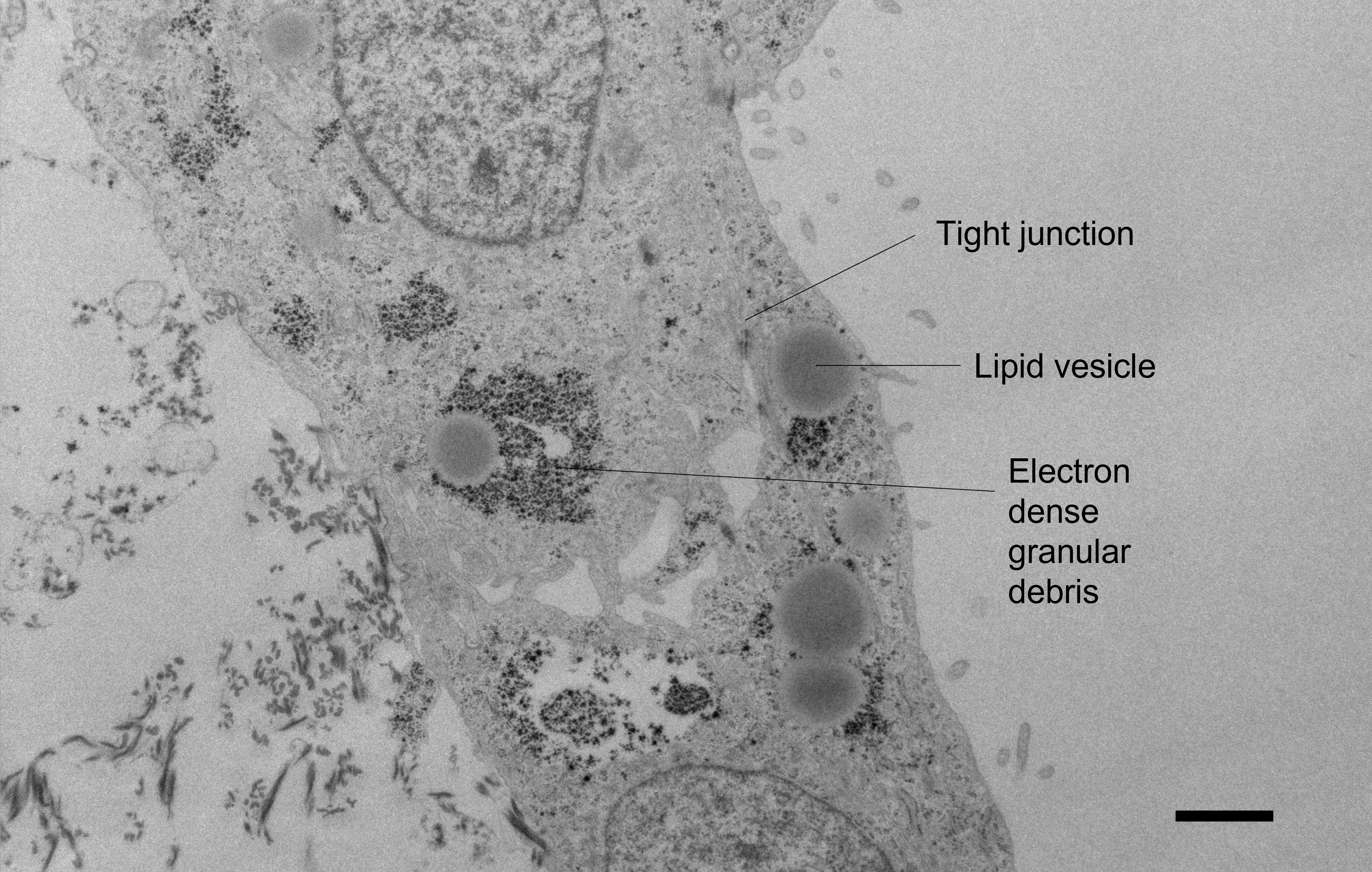

Supplement: S1 Fig — Transverse section showing the lipid vesicles and electron-dense granular material, which is progressively lost as the cells differentiate into endothelium. Scale bar = 1μm. (TIF) [file pone.0283954.s001.tif]

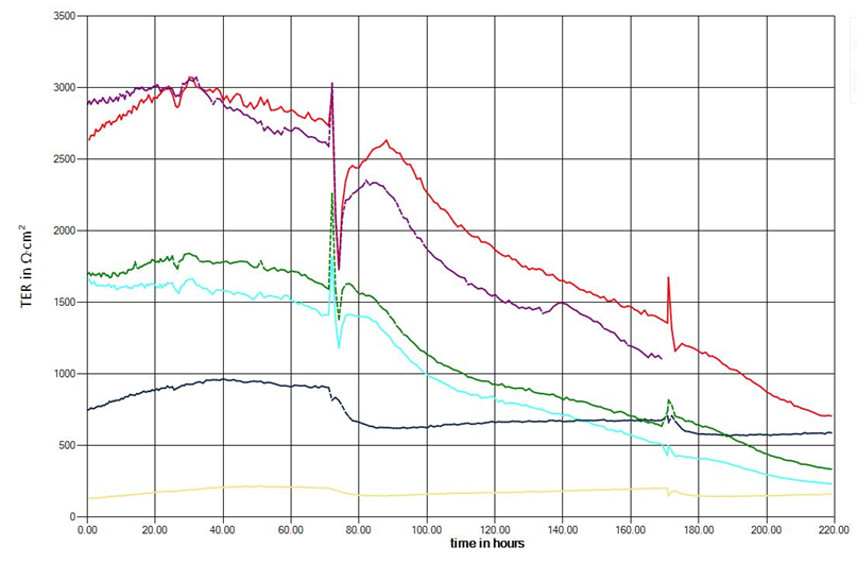

Supplement: S2 Fig — Medium changes were made at 72hrs and 170hrs. A and B, cultures with antibiotic (Penicillin/streptomycin): C and D, cultures without antibiotic. E and F, Caco-2 cells, cultured in parallel. (TIF) [file pone.0283954.s002.tif]

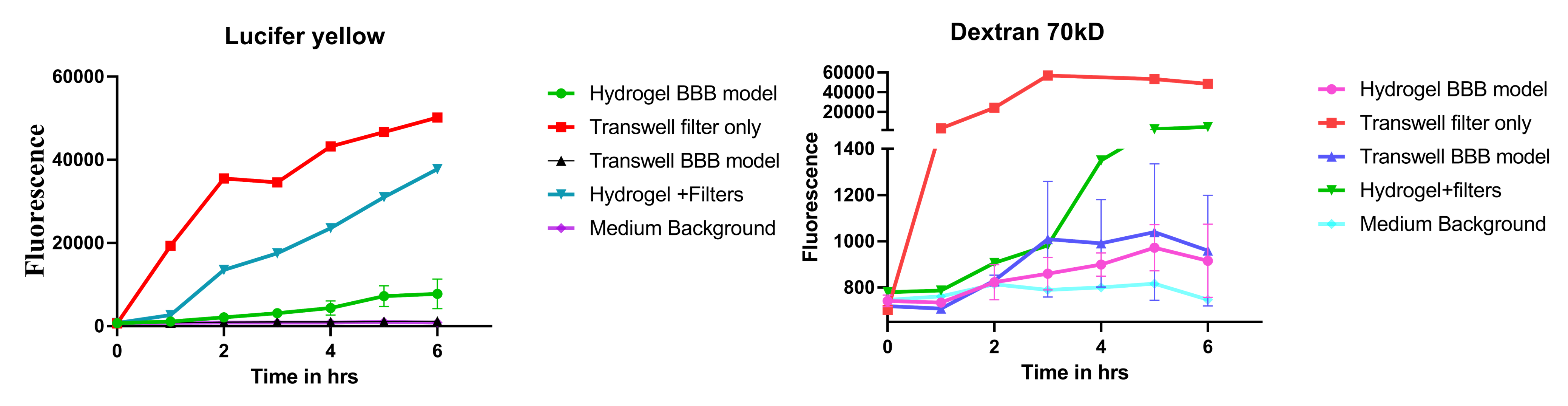

Supplement: S3 Fig — Matched cells on transwell filters only and transwell filter only are also shown. The background fluorescence released from the hydrogels is show as ‘medium background’ ie with no tracer. This type of plot was used to derive the permeability values of the paracellular tracers. (TIF) [file pone.0283954.s003.tif]

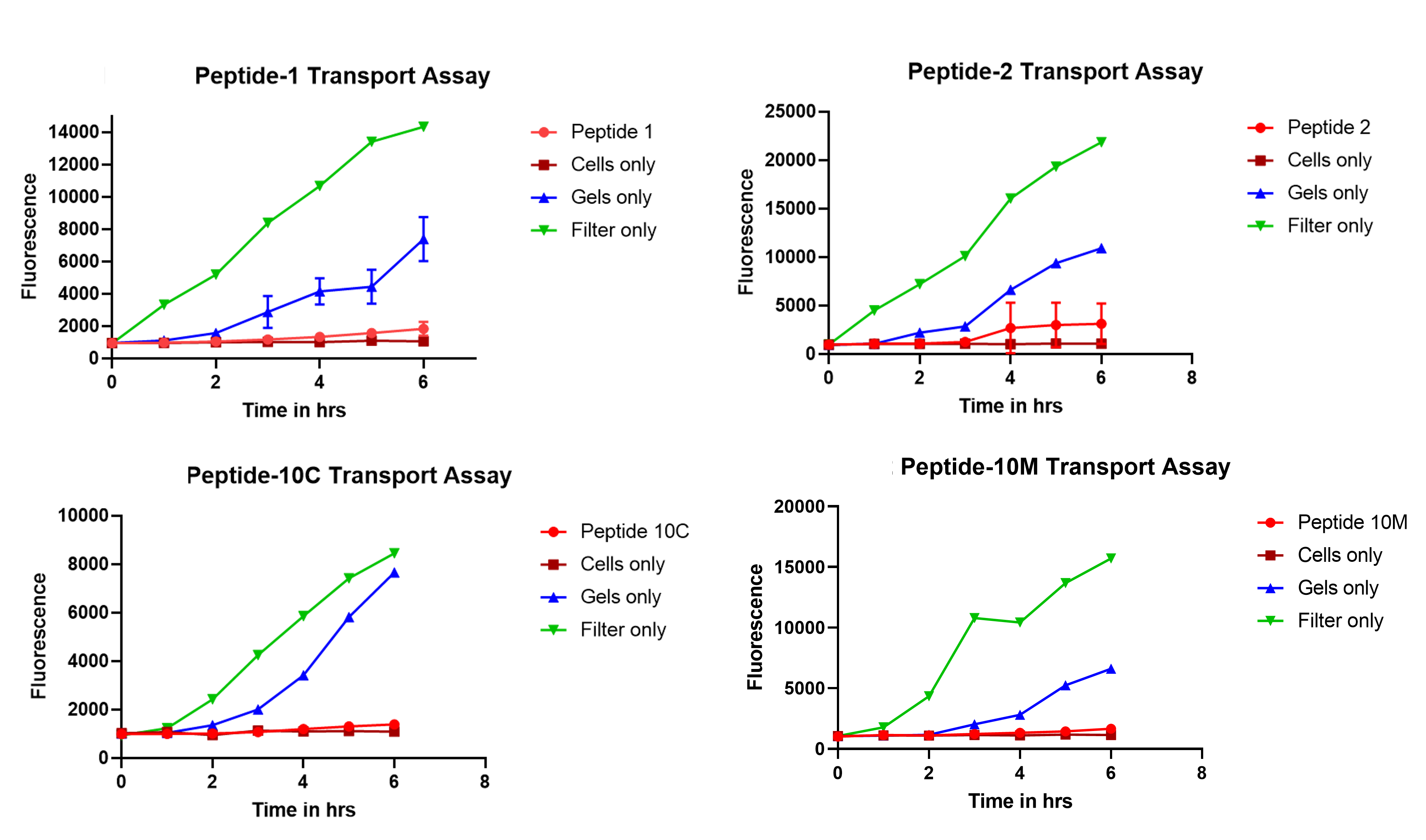

Supplement: S4 Fig — Matched endothelial cells on transwells (Cells only) compared with transwell filter only are also shown. (TIF) [file pone.0283954.s004.tif]

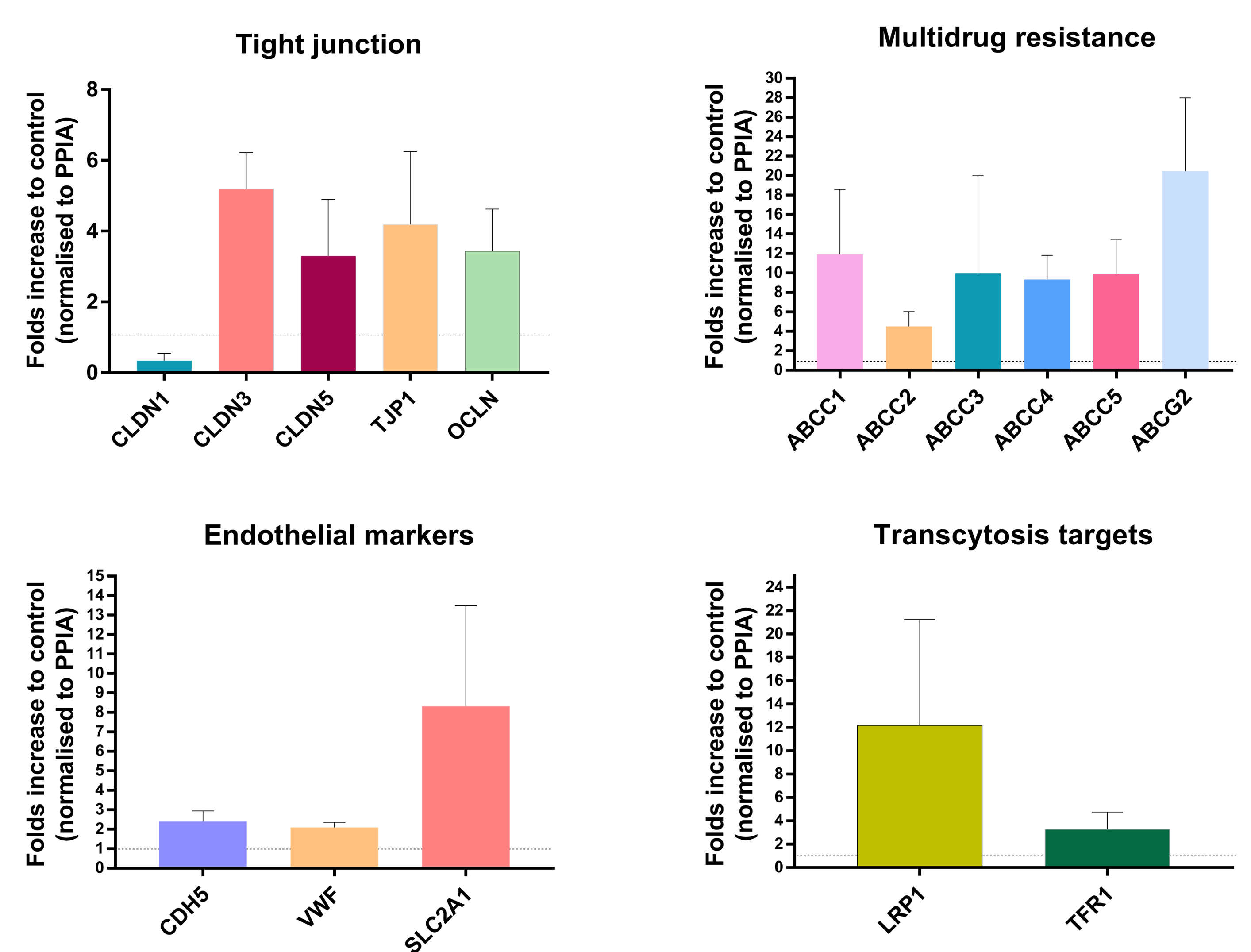

Supplement: S5 Fig — Expression in the differentiated endothelium was measured by qPCR and the level of expression compared with pre-differentiated hiPSCs (= 1). Values show mean and standard deviation from 4 preparations. (TIF) [file pone.0283954.s005.tif]
