# Supplementary material for: A hydrogel model of the human blood-brain barrier using differentiated stem cells
Source: PLoS One. 2023 Apr 4;18(4):e0283954. doi: 10.1371/journal.pone.0283954 (PMC10072488; doi:10.1371/journal.pone.0283954)
Supplement: Attachment — Submitted filename: Response to reviewers.docx [file pone.0283954.s006.docx]

We thank the reviewers for their careful consideration of the manuscript and have made the amendments indicated below. Each original comment is followed by an explanation and/or an outline or what changes have been made to the manuscript.

Major comments:

1. Fig. 1 is very limited and needs to be improved to increase the reproducibility for the reader (especially the part related to the differentiation of human iPSCs to endothelial-like cells). The key model components are listed to the right of the Transwell system; however, this overview of the primary components is not accompanied by arrows to point out their placement (please see the following papers with detailed workflow examples: doi: 10.3791/61629.; doi: 10.1080/10837450.2021.1872624). All used cell types should be visualized. Besides, the Figure 1 legend should include the description of critical steps as well as full names for abbreviations used in this Figure.

The figure has been replaced with a new figure with more detailed information and an extended figure legend describing the critical steps.

2. In the Results, the authors made the following statement: "The cells had weak junctional expression of the general endothelial marker VE-cadherin and weak expression of PECAM (CD31)…'' (p. 15, lines 4-6 from the top). How the "weakness" of the fluorescence signal coming from CD31 was measured? There was no quantitative analysis performed for this conclusion. Besides, the authors did not indicate whether the immunofluorescence staining of cultured cells was performed on glass coverslips or permeable Transwell inserts. If the latter applies, the insert membrane material (polycarbonate or polyester) can play a significant role in the visualization of junctional proteins (please see the paper doi: 10.12659/msmbr.900656.) and therefore could potentially explain why CD31 staining looks not as sharp as the other obtained images.

The immunofluorescence was carried out after differentiation of the cells, on chamber slides by confocal microscopy at day 10 of differentiation. This has now been detailed in the M&M section 2.6.

3. In the Results, the authors made the following statement: "The cells also had structures corresponding to Weibel Palade bodies, which appear in TEM images as bundles of fine tubules approximately 0.1μm thick and up to 3μm in length [22]." (p. 15, lines 8-10 from the top). Did the authors measure these parameters or these numbers are coming from reference #22? There should be an indication of software or technique used for the quantification of morphological structures on TEM images.

The characteristics of the Weibel-Palade body are as described in the original paper by Weibel and Palade (Ref 22). The measurement of size of the corresponding structures in our TEM images was made using the inbuilt software (scale-bars) on the JEM 1400 Microscope as described in section 2.7. This has been clarified in the Results section 3.1.

4. The representation of information in Figures and Tables needs a revision. Too many "short" figures may obscure the important results. It would be better to group all TEER data (measured with both cellZscope and Endohm meter) and paracellular permeability into a multi-panel figure (those with parts A, B, C, D, etc.). The same strategy can be applied to combining Figures 3 and 4 into a multi-panel figure. Therefore, Figures S4.1 – S4.4 should be combined as one main figure for part 3.3 of the Results. Similarly, Suppl. Figures S5.1-S5.4 should be combined and represented as the main figure for part 3.5. Parts 3.3 and 3.5 of the Results do not have any main figures, which makes the reader question what makes these findings less important. Transforming Tables 2 and 5 into bar graphs would be a much better visualization for comparing values between groups of data. Table 4, in turn, can be transformed into a Supplementary Figure.

Figures 3 and 4 have been combined into a two-part figure. (The following figure numbers are changed from the original, but the notes refer to the original numbering.)

We originally assembled the figures in their correct multi-part formats but also provided the individual panels as tif files as requested by the journal. It appears that the referee viewed the individual panels separately and therefore may not have seen the assembled figures as intended. We have now combined the multipart figures S3, S4 and S5 as single tif files. Because of the variability of the qPCR results, (explained by the variable rates of differentiation), we consider figure S5 to be more appropriate in the supplementary material.

The amount of data in tables 2, 3 and 4 is relatively small, If converted into bar graphs, there would only be 2-4 bars on each figure. As this referee has criticised ‘short figures’, providing this data as bar charts would produce additional ‘short figures’.

Table 4 (main paper) is the summary / collation of all permeability data, where the basic plots are shown in figure S4 (supplementary data). We think it is appropriate to have the summary data in the main paper and the basic data in the supplementary material, rather than the other way round.

5. It would be more appropriate to create a subsection entitled "Statistical Analysis" for the Materials and Methods instead of quite specific "Data analysis of permeability and transport assays". Does this mean the other data (qPCR and transporter activity) did not undergo statistical analysis? Why did authors use "one way ANOVA followed if appropriate by unpaired t-test" for data comparison instead of a one-way ANOVA followed by Tukey's post-hoc test?

The statistical analysis has been separated into a new section (2.13) in the M&M section with some additional clarification of tests used.

6. It looks like Fig. 7, Fig. S3, and Fig. S4 have only one sample per group or each time point. The sample size should be increased to run a statistical analysis or make the conclusions.

Figure 7 (now fig. 6) has bars showing SEM. This is stated in the legend, together with values of n and P. Errors bars are also shown on figure S3 for the hydrogels. Leakage across the filters alone (no hydrogel) are given as background information only. On figure S4, each permeability plot is formed by 7 data points. The slope of this plot constitutes one determination and n=4 determinations are assembled into the collated data in table 4.

7. The limitations of the developed model should be added to the Discussion.

A summary of the advantages and disadvantages of the model were given in the final paragraph of the discussion. Following the comment above and those of referee-2, we have extended this paragraph to give a more detailed critique of the model.

Minor comments:

1. In the Introduction, the authors said that "Hence, one desirable characteristic of a BBB model is the expression of continuous tight junctions, a high TEER and corresponding low permeability for paracellular tracers". It would be better to say "characteristics" since these are different BBB model aspects.

Corrected

2. It is not very clear why BBB models need the incorporation of a hydrogel. It should be better described in the Introduction.
3. What is the advantage of using astrocytes in the developed BBB model?

These points are also made by referee -2 - explanations have been added to the introduction.

4. Part 2.6. "Characterization of brain endothelial-like cells derived from human- induced pluripotent cells" should be replaced with a more specific title, for example, "Immunofluorescence staining". What kind of microscopy was used for Immunofluorescence staining imaging?

Changes made as indicated.

5. P. 22, line 14: "The second modification of the basic protocol was preselection of high adherence cells.". Did the authors mean their previously published protocol?

Reference added to clarify.

6. Please examine if all used abbreviatures in the text are kept consistently. For example, Line 3 in 2.2. Cell selection before seeding states "On day 8 after shift to UC medium, the cells were detached with accutase…". Did you mean UM? The previous paragraph states that "the cells were shifted to unconditioned media (UM) for 6 days". Corrected

7. Please revise the manuscript for minor typographical and grammatical errors. For example, the dots are missing between the sentences at line 15 of page 3 (Introduction) and line 9, of page 6 (Part 2.1).

Checked and corrected.

8. All images presented in Fig. 2 have two colors. Does the blue color in the nucleus represent DAPI staining? Please specify colors.

Amendment made to the legend of figure 2

9. Supplementary Fig. 1 has no arrows pointing out individual morphological structures or a scale bar.

The original figure had labels but they were not present on the tif image. They have now been added to the tif image.

10. Sample size (n) and data expression such as Mean ± SEM need to be added to the figures with TEER and transport assay data.

Each Cellzscope TEER trace (Fig 5 and Fig S2) comes from a single hydrogel or transwell culture. This is the nature of this type of data. The transport assays (Figs 6 and 7) show mean, SEM sample sizes and the legends give P values or statistical marking.

11. Please add lines with asterisks to denote statistical significance on Suppl. Figures S5.1-S5.4.

Because the data is variable between cultures (see above), we could not make any meaningful comparisons between levels of gene expression (which would not be valid anyway, due to variation in the efficiency of qPCR for different genes) or between the early and late stages of differentiation.

Reviewer #2: 

1. There are several publications in the scientific literature that describe the use of hIPSCs derived brain endothelial like cells. Based on the authors' data, it is not clear as to how their model system is more advantageous or more novel than these other published in vitro models utilizing hIPSCs derived brain endothelial like cells.

This point is also made by referee No. 1 and additional explanation of the advantages of hydrogel models has been added to the introduction. Discussion on the specific advantages and limitations of the hydrogel model described here have been added to the discussion.

2. Indeed, it is well known that other cell types of the neurovascular unit (i.e., astrocytes) can regulate the endothelium phenotypes in terms of barrier properties and transporter expression regulation. The authors described the use of astrocyte. However, the “general beneficial” effects of astrocytes on the barrier model are not described or discussed. In addition, the astrocytes have not been characterized, and the source of them is missing in the method section. Could you discuss on how the astrocytes potentially affect the monolayers? It has been used but why this specific cell type and how could it affect the cultures?

There is already a short statement in the discussion, that the astrocytes did not affect the barrier properties of the endothelium measured in this model. The paragraph has been expanded slightly to emphasize the point.

Information on the astrocytes has been added to the M&M section 2.4.

3. The authors should re-consider the permeability calculations. From the figures in the supplementary, it can be seen that the steady-state fluxes were not constant during the time course of the transport experiment. The authors should consider adding a statement whether sink conditions were maintained, and also provide the equation for calculating the permeability.

The reason for non-steady state flux in the hydrogel model is related to the delay in diffusion across the hydrogel (see section 2.11). This is why it was necessary to use just the slope of the permeability curves between 2-6 hours for rapidly-transferred molecules as described in section 3.2, or single time point levels at 24hours for molecules that could not be detected in the less than 8 hours (section 2.11). The method for calculation of Pe is standard for these type of cultures on filters and is described fully in reference [20]. The method of sampling from the lower chamber is described in section 2. 9. – this is not a continuous sink-condition.

4. The protein expression levels of key targets identified in the study would add more valuable information to the functional aspects of these BBB markers or transporters assessed in the hydrogel model. That information may be a great addition to the current study as the authors are comparing different culture configurations; transwell vs. hydrogel.

We agree that (eg) Western blotting of cultures for phenotypic markers would add to the study, but the recovery of endothelial-like cells from the hydrogel surface in sufficient quantities presented difficulties, which could not be addressed within the time available.

In response to the editorial comment, we have removed the short section on IgG transport from the results and M&M sections. This data was not important for the focus of the paper.
